# Annotations

**Published:** 1902-09-27

**Authors:** 


### ANNOTATIONS.

### Birth Rates in Different Classes of Society.

Whatever may be the cause it seems pretty clear that the birth rate is higher among the poor than among the well to do. The casuist may say, perhaps, that it is because of the multitude of their children that some people remain poor, and it is no doubt true that in a state of society such as exists at present—in which family ties are considerably loosened and grown up children do not admit the obligation to maintain their parents which used to be accepted without question—the parents of large families are almost of necessity condemned to poverty. This, however, certainly does not entirely, nor indeed principally account for the existing disproportion between the birth rates of different classes of society. It seems that there are over four times as many births, proportionately, in St. Luke's as in St. George's, Hanover Square, and that so far as poverty may be taken to express failure in life the nation is entrusting to its failures the important duty of carrying on the race. We are, however, by no means sure that this properly expresses the facts of the case. What makes for national greatness is not that calculating selfishness which leads young people to refrain from marriage until they can marry comfortably, surrounded with all the little luxuries in the midst of which they have been brought up; and still less is it to be found among those young couples who by a conspiracy of worldly-minded self indulgence voluntarily limit the number of their offspring. So long as courage, recklessness, intolerance of restrain, strong desires and indifference to the discomfort entailed in gratifying them remain active agents in the maintenance of freedom and the making of nations, we need not be over-concerned at the fact that the mean, the selfish and the calculating among us are not too quickly propagating their kind.

### The Mystery of Life.

The mystery of life is rendered more mysterious than ever by the observations which have been made of late concerning the behaviour of certain living organisms under the influence of extreme cold when, as Professor Dewar pointed out in his recent address at Belfast, all such chemical activities as are concerned with vital functions must be in abeyance. Bacteria have been kept at the temperature of liquid air for 20 hours, but their vitality was not affected, their functional activities remained unimpaired, and the cultures they yielded were normal in every respect. The same result was obtained when liquid hydrogen was substituted for liquid air. Seeds have been frozen for over 100 hours in liquid air, with no other result than to affect their protoplasm with a certain inertness from which it recovered with warmth. A research by Professor Macfadyen has shown that many varieties of micro-organisms can be exposed to the temperature of liquid air for a period of six months without any appreciable loss of vitality, although at such a temperature the ordinary chemical processes of the cell must cease. Thus we are brought up at once against a serious question, for although all chemical change was certainly in abeyance at the temperatures to which the cells were exposed, yet life persisted. Is life, then, dependent on structure? and so long as structure is maintained, can it take up the thread again where it was left off? or is life something quite outside physical law which, so long as the instrument on which it plays remains intact, can continue the same tune when once the instrument is unfrozen. The idea that life depended upon continued chemical action, and that although such action in the seed was slow it still went on, has for long seemed to many to be a comforting hypothesis, bringing vital action into line with other manifestations of the physical forces. But that life can stand still, that the whole process can be interrupted, that the living body can be brought down to the inertness of dead matter, and that then with the re-application of warmth it can go on again as before, is a startling proposition, and drives us to the conclusion that, after all, life is not entirely a matter of chemical change. Whether structure is the keynote; whether, like a watch, protoplasm will "go" when wound up; and whether heat is the winder, are deeper questions which at present are incapable of solution.

### The Cream of the Workers.

With the steady extension of factory legislation and of the machinery for enforcing its provision, it becomes a matter of no small importance that we should be quite clear that all such legislation is laid down upon the right lines; and this is not in every case quite as certain as some may think. That laws should be so contrived as to insure to the worker the best attainable surroundings, and the elimination of all avoidable risks, is surely right. Factory legislation, however, has not stopped here, but has attempted to improve the healthiness of certain trades, not only by the perfectly legitimate method of removing all injurious conditions of labour and penalising injurious processes, but by the much more questionable method of eliminating from these trades and occupations those who are likely to suffer from them. That a great saving of health is thus effected goes without saying. To take extreme cases, it is clear enough that much disease is prevented by turning out of the lead industries those anæmic girls who are peculiarly prone to suffer from plumbism, or by turning adrift out of the match-making trade all those who have bad teeth—a recognised antecedent to phosphorus poisoning; and so far as these industries are concerned, and their reputation for healthiness goes, nothing but good can result from such proceedings. Nevertheless, it is evident that by demanding certificates of fitness for employment, and by continually rejecting all but the healthiest, these inspected occupations do in fact skim the cream off the labour market, and although, as a matter of disease prevention, the obligation of obtaining certificates of fitness before entering upon certain forms of employment is undoubtedly effective, we cannot but feel that factory legislation is better employed in eliminating dangerous processes than in eliminating those who are unable to withstand the perils of these processes; in making trades healthy rather than in restricting admission to them to the cream of the workers.

